# Inhibition of the Functional Interplay between Endoplasmic Reticulum (ER) Oxidoreduclin-1α (Ero1α) and Protein-disulfide Isomerase (PDI) by the Endocrine Disruptor Bisphenol A*

**DOI:** 10.1074/jbc.M114.564104

**Published:** 2014-08-13

**Authors:** Masaki Okumura, Hiroshi Kadokura, Shoko Hashimoto, Katsuhide Yutani, Shingo Kanemura, Takaaki Hikima, Yuji Hidaka, Len Ito, Kohei Shiba, Shoji Masui, Daiki Imai, Susumu Imaoka, Hiroshi Yamaguchi, Kenji Inaba

**Affiliations:** From the ‡ School of Science and Technology, Kwansei Gakuin University, Gakuen 2-1, Sanda, Hyogo 669-1337, Japan,; the § Division of Protein Chemistry, Medical Institute of Bioregulation, Kyushu University, 3-1-1 Maidashi, Higashi-ku, Fukuoka 812-8582, Japan,; the ¶ RIKEN SPring-8 Center, RIKEN, 1-1-1 Kouto, Sayo-cho, Sayo-gun, Hyogo 679-5148, Japan,; the ‖ School Faculty of Science and Engineering, Kinki University, Kowakae 3-4-1, Higashi-Osaka, Osaka 577-8502, Japan,; the ‡‡ ProCube Business Division, Sysmex Corporation, 1-1-2, Murotani, Nishi-ku, Kobe, Hyogo, 651-2241, Japan, and; the ** Institute of Multidisciplinary Research for Advanced Materials, Tohoku University, Katahira 2-1-1, Aoba-ku, Sendai 980-8577, Japan

**Keywords:** Endoplasmic Reticulum (ER), Enzyme Inhibitor, Protein Conformation, Protein Folding, Protein Isomerase

## Abstract

Bisphenol A (BPA) is an endocrine disruptor that may have adverse effects on human health. We recently isolated protein-disulfide isomerase (PDI) as a BPA-binding protein from rat brain homogenates and found that BPA markedly inhibited PDI activity. To elucidate mechanisms of this inhibition, detailed structural, biophysical, and functional analyses of PDI were performed in the presence of BPA. BPA binding to PDI induced significant rearrangement of the N-terminal thioredoxin domain of PDI, resulting in more compact overall structure. This conformational change led to closure of the substrate-binding pocket in b′ domain, preventing PDI from binding to unfolded proteins. The b′ domain also plays an essential role in the interplay between PDI and ER oxidoreduclin 1α (Ero1α), a flavoenzyme responsible for reoxidation of PDI. We show that BPA inhibited Ero1α-catalyzed PDI oxidation presumably by inhibiting the interaction between the b′ domain of PDI and Ero1α; the phenol groups of BPA probably compete with a highly conserved tryptophan residue, located in the protruding β-hairpin of Ero1α, for binding to PDI. Consistently, BPA slowed down the reoxidation of PDI and caused the reduction of PDI in HeLa cells, indicating that BPA has a great impact on the redox homeostasis of PDI within cells. However, BPA had no effect on the interaction between PDI and peroxiredoxin-4 (Prx4), another PDI family oxidase, suggesting that the interaction between Prx4 and PDI is different from that of Ero1α and PDI. These results indicate that BPA, a widely distributed and potentially harmful chemical, inhibits Ero1-PDI-mediated disulfide bond formation.

## Teaser

**Background:** Protein-disulfide isomerase (PDI) has previously been identified to bind bisphenol A (BPA), an endocrine disrupter.

**Results:** BPA inhibited Ero1α-PDI-mediated disulfide bond formation.

**Conclusion:** BPA significantly inhibited the Ero1α and PDI oxidative cycle, probably through closure of the substrate- and Ero1α-binding pocket in the PDI **b′** domain.

**Significance:** BPA may have inhibitory effects on oxidative folding of secretory and membrane proteins.

## Introduction

**FIGURE 1.**
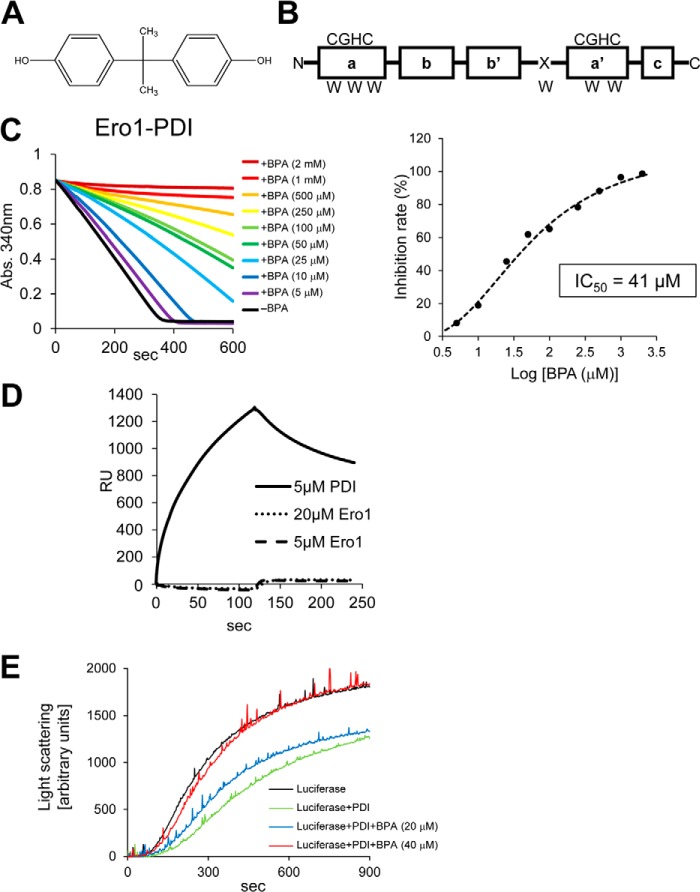
**Inhibition of Ero1α by BPA.** *A*, chemical structure of BPA. *B*, domain organization of PDI_full-length_. The four thioredoxin domains, which are designated **a**, **b**, **b′**, and **a′**, near the N terminus and the C-terminal α-helical domain **c′** are shown in *boxes*. Active site sequences (*CGHC*) and tryptophan residues (*W*) are also indicated. *C*, *left*, oxidative activity of Ero1α (4 μm) toward PDI (10 μm) in the presence of various concentrations of BPA was assessed by measuring the decrease in *A*_340_. This is due to NADPH consumption mediated by glutathione reductase in a buffer containing GSH and PDI. *Right*, plot of percentage inhibition as a function of BPA concentration determined from the initial velocity of the decrease in *A*_340_. *D*, detection of BPA binding to Ero1α and PDI by SPR. *E*, chaperone activity of PDI in the presence of BPA (20 or 40 μm). The chaperone activity of PDI was analyzed using luciferase as a model substrate.

Bisphenol A (BPA^4^; 2,2′-bis(4-hydroxyphenyl) propane) is an estrogenic endocrine-disrupting chemical widely used in the manufacture of plastics (1). BPA competes with several steroid ligands for binding to steroid receptors due to its structural similarity to steroids, and hence it inhibits or sometimes stimulates physiological functions, such as tumor cell proliferation (2, 3). BPA is a metabolically recalcitrant organic compound (Fig. 1*A*) and accumulates in tissues once taken up by living organisms. In addition to its antagonist/agonist effects on steroid hormone receptors, BPA induces a number of detrimental effects on the central nervous system, including induction of abnormal behavior in mice and morphogenic malformation in *Xenopus laevis* (4). Several lines of evidence demonstrate that BPA exposure causes adverse effects on human health (5).

Recently, we isolated a BPA-binding protein from rat brain extracts, which was identified as the canonical protein-disulfide isomerase (PDI) (6). PDI, an oxidoreductase abundantly present in the endoplasmic reticulum (ER), plays a central role in the folding of secretory proteins by catalyzing disulfide bond formation and preventing aggregation of unfolded or misfolded proteins (7, 8). PDI consists of four thioredoxin domains, termed **a**, **b**, **b′**, and **a′**, from the N terminus and an additional ill defined α-helical **c** domain located at the C terminus (9, 10) (Fig. 1*B*). The **a** and **a′** domains contain a catalytic active site composed of a CGHC sequence, whereas the central domains, **b** and **b′**, possess hydrophobic surfaces responsible for the binding of incompletely folded protein substrates (11–15). Additionally, PDI contains an **x**-linker region between the **b′** and **a′** domains, which may function as a gate for access to the substrate-binding site of the **b′** domain (10, 16, 17).

During disulfide bond formation (18, 19), Ero1 enzymes generate a protein disulfide bond *de novo* consuming an oxygen molecule as an electron acceptor and transfer it to PDI, resulting in oxidation of PDI (20, 21). To react specifically with PDI, a protruding β-hairpin loop of Ero1α binds a hydrophobic pocket in the PDI **b′** domain (22, 23). Because Ero1 family enzymes yield hydrogen peroxide, a source of reactive oxygen species, as a result of the generation of protein disulfide bonds, their PDI oxidation activity must be tightly regulated in response to the redox environment of the ER. Regulatory mechanisms for the Ero1α-PDI oxidative cycle have been studied in detail and are outlined in several review articles (19–21, 24).

We previously found that BPA binds the PDI **b′** domain and thereby inhibits the disulfide bond formation activity of PDI (25, 26). Because both Ero1α and BPA bind the PDI **b′** domain, we hypothesized that BPA may interfere with Ero1α-mediated oxidation of PDI. To gain further insights into the structural and functional effects of BPA on PDI, detailed biochemical and biophysical analyses of PDI were performed in the presence of BPA. Consequently, BPA binding significantly inhibited oxidative folding of bovine pancreas trypsin inhibitor (BPTI) catalyzed by Ero1α and PDI. BPA induced significant spatial rearrangement of the **a** and **b′** domains of PDI, leading to a more compact overall structure. Thus, BPA probably inhibits the Ero1-PDI disulfide bond formation pathway of the ER, an essential organelle engaged in the synthesis of secretory and membrane proteins.

## EXPERIMENTAL PROCEDURES

### 

#### 

##### Materials

BPA was purchased from Wako Pure Chemical Industries, Ltd. (Osaka, Japan). All other chemicals and solvents used were of the highest grade available.

##### Expression and Purification of PDI_full-length_, PDI(120–353), PDI(354–509), ERp46, Prx4, and Ero1α

Rat PDI cDNA was cloned as described previously (6). Plasmids used for expression of PDI_full-length_, PDI(120–353), and PDI(354–509) fragments were constructed as described previously (25). Plasmids for ERp46, Prx4, and Ero1α have been described previously (23, 27, 28). Each construct was overexpressed in *Escherichia coli*. Cells were harvested and homogenized. Recombinant proteins were then purified by a combination of several types of chromatography. The final preparation was subjected to SDS-PAGE, and the purity was confirmed to be over 90% for all recombinant proteins (data not shown) (29).

##### Circular Dichroism (CD) Spectroscopy

CD measurements were performed on a JASCO J-600 and J-720 spectropolarimeter (JASCO Corp., Tokyo, Japan). The CD spectra of PDI(120–353) and PDI(354–509) were measured in 20 mm phosphate buffer (pH 7.4) at room temperature, using a quartz cell with a 1.0-mm path length for far-UV (260–200 nm) and a 10-mm path-length for near-UV spectra (340–250 nm). Protein concentrations were as follows: for PDI_full-length_, 0.95 mg/ml in near-UV spectra and 0.24 mg/ml in far-UV spectra; for PDI(120–353), 0.69 mg/ml in near-UV spectra and 0.31 mg/ml in far-UV spectra; for PDI(354–509), 0.93 mg/ml in near-UV spectra and 0.31 mg/ml in far-UV spectra. All samples were centrifuged at 15,000 × *g* for 15 min, and then supernatants were used for measurements.

##### Differential Scanning Calorimetry (DSC)

DSC measurements were performed using a Microcal VP-capillary DSC platform (GE Healthcare) at a scan rate of 60 °C h^−1^, 120 °C h^−1^, and 200 °C h^−1^ for PDI(120–353) and 200 °C h^−1^ for PDI(354–509). Protein solutions were dialyzed against 20 mm phosphate buffer (pH 7.0) and thereafter centrifuged at 15,000 × *g* for 15 min. Supernatants were filtered through a 0.22-μm pore size membrane before measurement. Protein concentration of PDI(120–353) was 0.32 mg/ml for 200 °C h^−1^ and 0.97 mg/ml for 60 and 120 °C h^−1^. Protein concentration of PDI(354–509) was 0.32 mg/ml. Reversibility of heat denaturation for PDI(120–353) was performed at a scan rate of 60 °C h^−1^ in 20 mm phosphate buffer (pH 7.0) with a protein concentration of 0.72 mg/ml. Thermograms were integrated using ORIGIN software (Microcal Software, Northampton, MA) to estimate binding constants.

##### Dynamic Light Scattering (DLS)

The hydrodynamic sizes of PDI(120–353) and PDI(354–509) in solution were measured with a Zetasizer NanoZS (Malvern Instruments, Malvern, UK), based on the principle of dynamic light scattering (30). Briefly, hydrodynamic diameter was converted by the Stokes-Einstein equation. Polydispersity, or heterogeneity of Brownian motion, was calculated from the S.D. value of the distribution at each BPA concentration. All samples were centrifuged at 15,000 × *g* for 10 min, and the supernatants were filtered through a 0.22-μm pore size membrane before measurement. The protein concentration was 0.89 mg/ml for PDI(120–353) and 1.18 mg/ml for PDI(354–509).

##### Homology Modeling of PDI(120–353) and PDI(354–509)

To predict the structure of PDI(120–353) and PDI(354–509), a protein homology/analogy recognition engine (Phyre) was used (31).

##### Small Angle X-ray Scattering (SAXS)

SAXS data were collected on the BL45XU beamline at the RIKEN SPring-8 Center (Hyogo, Japan) (32). The x-ray wavelength was 1.0 Å, and a PILATUS 300K-W system (DECTRIS, Baden, Switzerland) was employed as a detector with a camera distance of ∼2,000 mm. Scattering intensity was recorded at 293.2 K with 18 continuous images, each with an exposure time of 10 s. Images were checked for radiation damage, and data thus selected were averaged. All protein solutions were prepared in 20 mm phosphate buffer (pH 7.0), and SAXS profiles for PDI(120–353) in the presence or absence of BPA (with a 1:1 molar ratio of PDI(120–353)/BPA) were collected in a concentration range of 1.5–4.0 mg/ml. SAXS profiles for bovine serum albumin (*M*_r_ of 66,400; Sigma-Aldrich) were also collected as a reference for determining the relative molecular weight of PDI(120–353). All samples were centrifuged at 15,000 × *g* for 15 min, and supernatants were used for measurements.

Small angle scattering profiles were fitted by Guinier approximation with the equation,

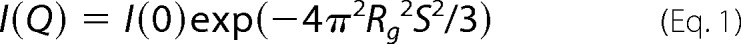
 where *I*(0) and *R_g_* are the forward scattering intensity at *Q* = 0 and the radius of gyration, respectively (33). The *I*(0) value is proportional to the averaged molecular weight and concentration. The pair distribution function, *P*(*r*), was calculated by indirect Fourier transformation using GNOM (34).

##### NADPH Consumption Assay

Glutathione reductase (1 unit) was incubated with NADPH (200 μm), reduced glutathione (300 μm), human PDI (10 μm), the hyperactive form, including mutations of C104A and C131A, of human Ero1α (4 μm) (27), and various concentrations of BPA (5–2000 μm). The oxidation of NADPH by glutathione reductase led to a decrease in absorbance at 340 nm (*A*_340_). A molar extinction coefficient of 6,200 m^−1^ cm^−1^ for NADPH was used for the calculations. The reaction was initiated by adding Ero1α. Changes in absorbance at 340 nm were monitored with a Hitachi U-3310 spectrophotometer at 30 °C in 50 mm Tris-HCl, pH 7.5, 0.3 m NaCl. IC_50_ values were calculated by curve fitting using ImageJ.

##### BPTI Folding Assay

Reduction and denaturation of BPTI (Takara Bio Inc.) were performed as described previously (27, 35–37). Fully denatured and reduced BPTI (30 μm) was refolded at 30 °C in oxygen-saturated buffer containing 50 mm Tris-HCl (pH 7.5), 300 mm NaCl, 1 μm PDI, a 1 μm concentration of the hyperactive form of Ero1α, and 40 μm BPA. For the Prx4-PDI- or glutathione-PDI-mediated folding of BPTI, the same procedure was used, but the buffer was degassed by flushing N_2_ gas. Reactions were performed at 30 °C in buffer containing 50 mm Tris-HCl (pH 7.5), 300 mm NaCl, 1 μm PDI, 0.1 μm Prx4, 200 μm H_2_O_2_, and 40 μm BPA (27, 28) or buffer containing 50 mm Tris-HCl (pH 7.5), 300 mm NaCl, 1 μm PDI, 2 mm GSH, 0.2 mm GSSG, and 40 μm BPA (38, 39). The resulting sample was analyzed by HPLC using a TSKgel Protein C4-300 column (4.6 × 150 mm; Tosoh Bioscience) at 229 nm, and identities of the peaks obtained were confirmed by MALDI-TOF/MS (Bruker), as described previously (28).

##### Chaperone Activity of PDI

The chaperone activity of PDI was measured using luciferase as a model substrate (40). To monitor effect of PDI on the thermal aggregation of luciferase, the substrate protein was diluted to a final concentration of 2 μm in buffer containing 40 mm HEPES-KOH, pH 7.5, PDI (4 μm) and BPA (20 or 40 μm) under constant stirring and incubated at 45 °C during the measurement. Thermal aggregation was monitored by measuring light scattering using a Hitachi Spectrofluorometer F2500. The wavelength of light entered and monitored was 350 nm.

##### Analyzing the Oxidation Status of PDI in HeLa Cells

HeLa cells were maintained in Dulbecco's modified Eagle's medium (DMEM) (Nacalai Tesque) supplemented with 10% fetal bovine serum at 37 °C under 5% CO_2_. Where indicated, cells were treated with 10 mm dithiothreitol (DTT), 1 mm diamide, or the indicated concentrations of BPA for the indicated times before preparing samples. To analyze the redox state of PDI in cells, cells were washed twice with phosphate-buffered saline (PBS) and treated with ice-cold 10% trichloroacetic acid. After a 20-min incubation on ice, cellular proteins were collected by centrifugation, washed once with ice-cold acetone to remove acid, and dissolved in alkylation buffer (100 mm Tris-HCl (pH 6.8), 2% SDS) containing 5.5 mm PEG-maleimide 2000 (SUNBRIGHT ME-020MA, NOF) and protease inhibitors (10 μg/ml pepstatin A, 1 mm benzamide, and 1 mm phenylmethylsulfonyl fluoride) (41). The samples were then agitated for 20 min and incubated for another 30 min at 37 °C to obtain PEG-maleimide 2000-treated cell lysates. Proteins from the resulting cell lysates were separated by 10% SDS-polyacrylamide gel electrophoresis, blotted onto an Immobilon-P membrane (Millipore), incubated with rabbit α-PDI antiserum (27) and with an appropriate secondary antibody, and detected with ECL Select Western blotting Detection Reagent (GE Healthcare) and LAS-3000 mini (Fujifilm) to visualize the oxidation status of PDI in the cells.

##### DTT Pulse Experiment

To study the effect of BPA on the reoxidation of PDI in HeLa cells, the culture of HeLa cells was treated with 10 mm DTT for 10 min to reduce PDI (pulse) (42). DTT was then removed by washing the cells twice with fresh medium containing the indicated concentrations of BPA. The cultures of the resulting washed cells were then grown in the fresh medium for 10 min to allow the reoxidation of PDI (chase). The chase was performed in the presence of BPA to study the effect of BPA on the reoxidation of PDI. The cultures were then subjected to alkylation with PEG-maleimide 2000 and subsequent immunoblotting with anti-PDI antiserum.

## RESULTS

### 

#### 

##### Inhibition of the Ero1α-PDI Oxidative Cycle by BPA in Vitro

To determine the rate of Ero1α-mediated PDI oxidation, we previously developed an NADPH consumption assay, in which oxidation of NADPH is coupled to Ero1α-mediated catalysis of PDI oxidation through the intermediary actions of glutathione reductase and glutathione (27). In this assay, the rate of Ero1α-mediated PDI oxidation was compared by measuring the initial velocity of the decrease in *A*_340_ due to the consumption of NADPH (Fig. 1*C*, *left*). BPA markedly inhibited Ero1α-mediated catalysis of PDI oxidation in a dose-dependent manner, with an IC_50_ of 41 μm (Fig. 1*C, right*). To examine whether BPA acts on PDI or Ero1α in exerting this inhibitory effect, we analyzed the binding of BPA to PDI or Ero1α by surface plasmon resonance (SPR). We observed that BPA directly associated with PDI but not with Ero1α (Fig. 1*D*). Hiroi *et al.* (6) indicated that the affinity of BPA for PDI was 22.6 μm, which is almost comparable with the IC_50_ value estimated by the PDI oxidation assay (Fig. 1*C*). It is conceivable that BPA inhibits Ero1α-mediated catalysis of PDI oxidation by binding a site on PDI critical for functional interplay with Ero1α. We next investigated the effect of BPA on the chaperone activity of PDI using luciferase as a model substrate (40). Luciferase aggregated, taking hundreds of seconds at 45.0 °C (Fig. 1*E*). Although the presence of PDI prevented the luciferase aggregation to a significant extent, the addition of BPA again caused the aggregation in a dose-dependent manner. Thus, BPA has an inhibitory effect on the chaperone activity of PDI.

##### Inhibitory Effect of BPA on Oxidative Protein Folding Catalyzed by the Ero1α-PDI Pathway

**FIGURE 2.**
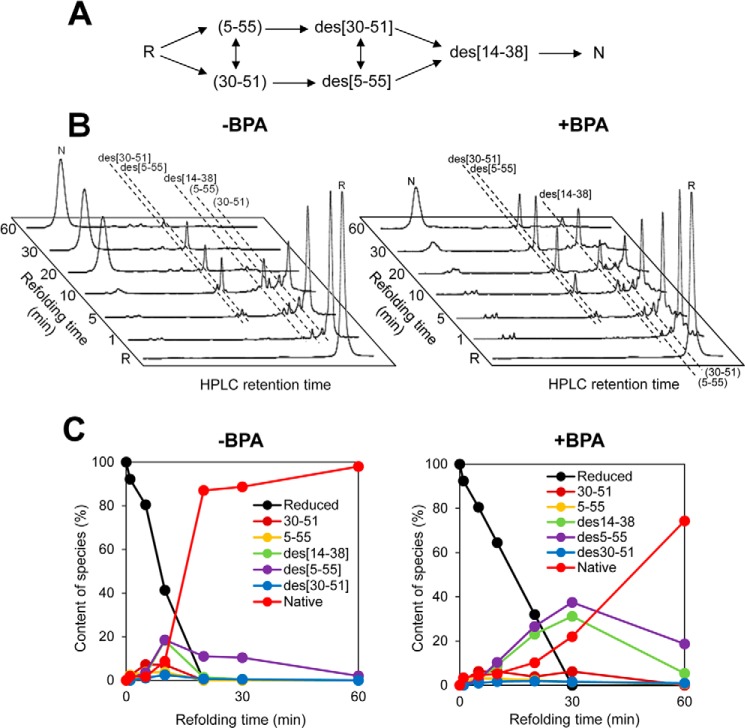
**Ero1α-PDI-driven oxidative folding of BPTI in the presence of BPA.** *A*, schematic representation of the oxidative folding pathways of BPTI in the presence of glutathione. *R* and *N* represent reduced and native form of BPTI (5–55, 14–38, and 30–51). The disulfide parings of folding intermediates at each step are shown in *parentheses*, and the missing disulfide is indicated with the prefix “des.” *B*, HPLC profiles indicating the time course of oxidative folding of BPTI catalyzed by Ero1α-PDI in the absence (*left*) and presence (*right*) of BPA. Folding was initiated by the addition of Ero1α and PDI to reduced BPTI (30 μm) in a buffer saturated with molecular oxygen (no thiol agents, such as glutathione, were present). At the indicated time points, the reaction was quenched by HCl and analyzed by HPLC. *C*, quantitative analysis of each folding species based on the HPLC profiles shown in *B*. The occupancy of each species was plotted as a function of refolding time.

**FIGURE 3.**
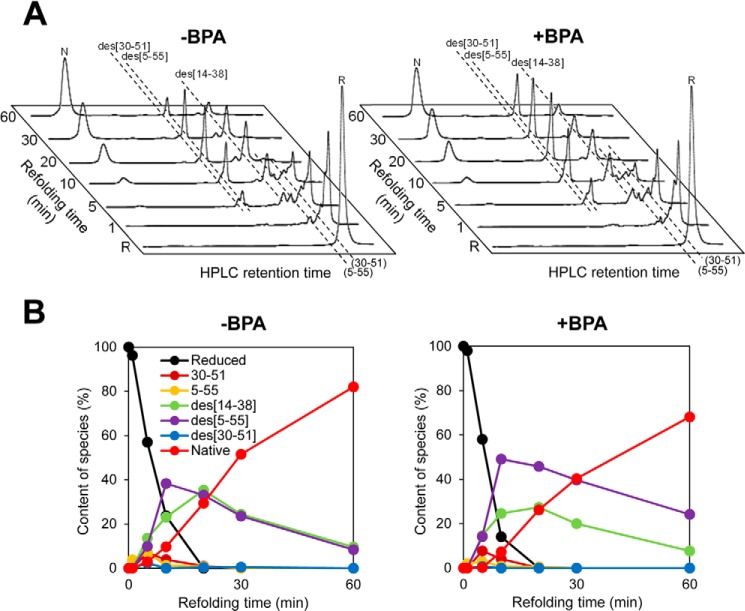
**Glutathione-PDI-mediated oxidative folding of BPTI in the presence of BPA.** *A*, HPLC profiles indicating the time course of oxidative folding of BPTI catalyzed by glutathione-PDI in the absence (*left*) and presence (*right*) of BPA. Folding was initiated by the addition of PDI to reduced BPTI (30 μm) in a degassed buffer containing 2 mm GSH and 0.2 mm GSSG (39). At the indicated time points, the reaction was quenched by HCl and analyzed by HPLC. *B*, quantitative analysis of each folding species based on the HPLC profiles shown in *A*. The occupancy of each species was plotted as a function of refolding time.

The results shown above suggest that BPA may inhibit oxidative protein folding catalyzed by Ero1α and PDI in combination. To test this possibility, we measured the oxidative folding of BPTI, as a model substrate, in the presence of Ero1α, PDI, saturated levels of oxygen, and BPA. BPTI contains six cysteine residues that participate in three native intramolecular disulfide bonds (Cys^5^–Cys^55^, Cys^14^–Cys^38^, and Cys^30^–Cys^51^). Glutathione-mediated oxidative folding pathways of BPTI have been well characterized by HPLC (Fig. 2*A*) (43, 44). The species R, species (5–55) and (30–51), des species (des(30–51), des(5–55), and des(14–38)), and N represent no disulfide bond, one disulfide bond, two disulfide bonds, and native disulfide bonds, respectively. As predicted, BPA substantially slowed down the generation of the N species (Fig. 2, *B* and *C*). Notably, whereas BPA inhibited both the early and late steps of BPTI folding, the effect was even more pronounced in the conversion of the des species to the N species than in the initial disulfide introduction during the early stage of folding. Previous in depth studies indicate that PDI plays a critical role in the formation of native disulfide bonds within partially structured and kinetically trapped folding intermediates, especially des species, of BPTI during the late steps of folding (7, 28). Likewise, the present glutathione-PDI-mediated BPTI refolding assay demonstrated that BPA inhibited slightly but significantly the conversion of kinetically trapped intermediates to a native state, probably due to the reduced disulfide rearrangement ability of BPA-bound PDI (Fig. 3).

**FIGURE 4.**
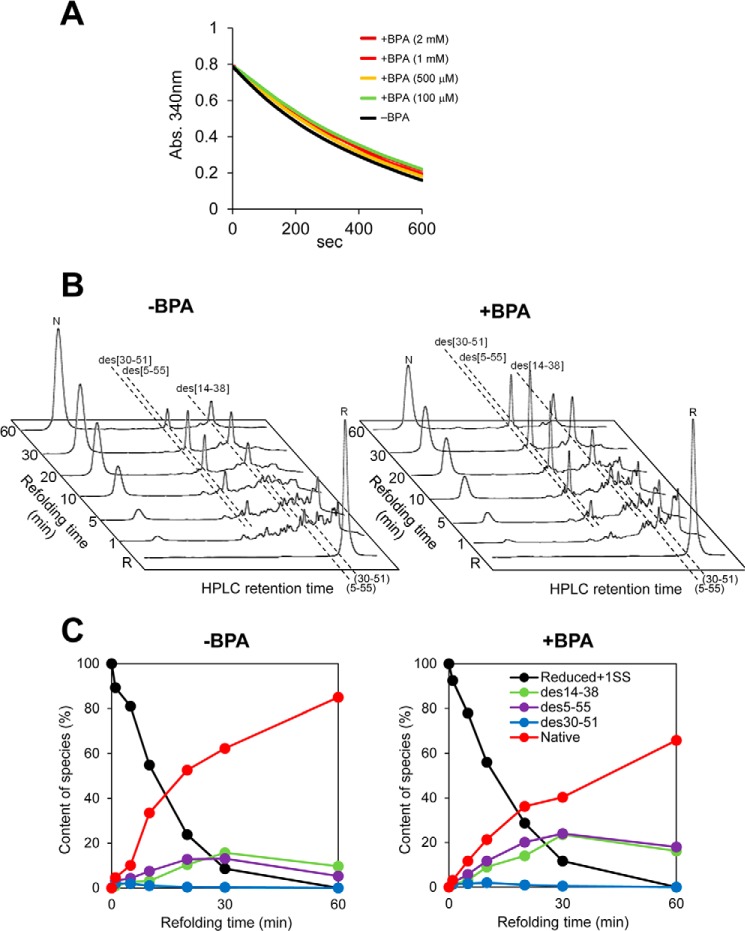
**Oxidative activity of Prx4 toward PDI in the presence of BPA.** *A*, oxidative activity of Prx4 (0.4 μm) toward PDI (10 μm) in the presence of various concentrations of BPA was assessed by measuring the decrease in *A*_340_. *B*, Prx4-PDI-mediated oxidative folding of BPTI. HPLC profiles indicate the time course of oxidative folding of BPTI catalyzed by Prx4-PDI in the absence (*left*) and presence (*right*) of BPA (40 μm). Folding was initiated by the addition of Prx4 (0.1 μm) and PDI (1 μm) to reduced BPTI (30 μm) in a buffer containing H_2_O_2_ (200 μm). At the indicated time points, reactions were quenched with HCl and analyzed by HPLC. GSH/GSSG redox reagents were not included in this assay. *C*, quantitative analysis of each folding species based on the HPLC profiles shown in *B*. The occupancy of each species was plotted as a function of refolding time.

Meanwhile, BPA had no effect on the Prx4-catalyzed PDI oxidation (Fig. 4*A*). In line with this, the early stage of BPTI folding catalyzed by Prx4-PDI pathway was little affected by the addition of BPA (Fig. 4*B*). However, the late steps of BPTI folding catalyzed by this pathway were significantly inhibited (Fig. 4*C*), as was the case with glutathione-PDI-mediated folding (Fig. 3*B*). We thus infer that BPA compromises the ability of PDI to recognize folding intermediates as well as the functional interplay between PDI and Ero1α.

##### BPA Retards the Reoxidation of PDI in HeLa Cells

Because BPA inhibited the ability of Ero1α to oxidize PDI *in vitro*, we envisaged that BPA might slow down the reoxidation of PDI in cells. To test this possibility, we first treated the cultures of HeLa cells with 10 mm DTT for 10 min to reduce PDI and washed the cells to remove DTT from the culture (42). We then cultivated the cells in a medium without DTT for 10 min to allow the reoxidation of PDI. This step was performed in the presence of various concentrations of BPA to study the effect of BPA on the reoxidation of PDI within the cells.

**FIGURE 5.**
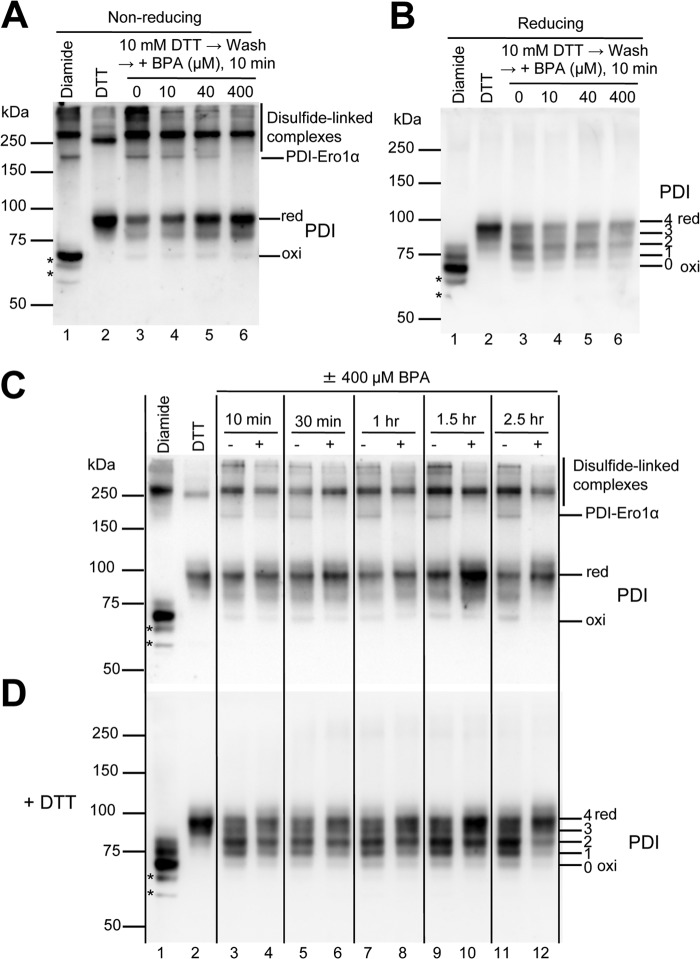
**BPA affects the redox state of PDI in HeLa cells.** *A* and *B*, the effect of BPA on the reoxidation of PDI was studied by cultivating DTT-pretreated HeLa cells in fresh medium without DTT in the presence of the indicated concentrations of BPA for 10 min (*lanes 3–6*). To visualize the oxidation status of PDI, cellular proteins were subjected to alkylation with PEG-maleimide 2000 and separation on SDS-PAGE under non-reducing conditions in *A* and reducing conditions in *B*, followed by immunoblotting with antibody to PDI. Each *lane* contains 5 μg of proteins from each lysate. The positions of the disulfide-linked complexes that involve PDI are indicated on the *right* in *A*. The band assignable to PDI-Ero1α complex, which was also recognized by antibody to Ero1α (not shown), is marked as *PDI-Ero1*α. To know the positions of the fully oxidized (*oxi*) and reduced (*red*) forms of PDI, cells were treated with 1 mm diamide and 10 mm DTT for 10 min, respectively, before alkylation of free cysteines (*lanes 1* and *2*). Note that some disulfide-linked complexes are refractory to these treatments (see *lanes 1* and *2* in *A*). The numbers of PEG-maleimide 2000 alkylation on the active site cysteines of PDI are indicated on the *right* in *B*. Note that the presence of PEG-maleimide 2000 in the sample caused slower migration of even the oxidized form of PDI on the gels when compared with PDI that was not treated with the alkylation reagent (not shown). We suggest that this could have been caused by the alkylation of two free non-catalytic cysteines on PDI (Cys^312^ and Cys^343^). Bands marked with an *asterisk* may represent some degradation products of PDI or nonspecific bands that appeared when the cultures were treated with diamide. *C* and *D*, to examine the effect of BPA on the redox state of PDI in cells, HeLa cells that had not been treated with DTT were incubated with or without 400 μm BPA for the indicated times. In *lanes 3*, *5*, *7*, and *9*, dimethyl sulfoxide (a solvent of BPA) was added to the culture to a final concentration of 0.2%. To visualize the redox state of PDI, the cellular proteins were alkylated with PEG-maleimide 2000 as described in the legend to *A*. In *D*, proteins were reduced with 50 mm DTT before electrophoresis. Note that BPA caused the accumulation of a large portion of PDI in its reduced form, in agreement with the *in vitro* finding that BPA inhibited the Ero1α-catalyzed oxidation of PDI.

To determine the oxidation (disulfide-bonded) status of PDI in the cells, we treated the cells directly with acid to prevent post-harvest oxidation of cysteines and then alkylated free cysteines with PEG-maleimide 2000 (average molecular mass, 2,300 Da). This modification stabilizes disulfide-linked complexes and retards the migration of the reduced form of proteins on a gel (41). The oxidation status of PDI in the cells was then visualized by immunoblotting using antibody to PDI (Fig. 5*A*). In addition to the oxidized and reduced forms of PDI, we detected a number of bands with different apparent molecular masses that reacted with antibody to PDI (Fig. 5*A*). We suggest that the latter bands represent disulfide-linked complexes between PDI and its partners for two reasons. First, they ran slower than the monomeric forms of PDI. Second, when the samples were treated with reducing agent before electrophoresis, these bands disappeared, giving rise to bands corresponding to the monomeric forms of this protein (Fig. 5*B*). Remarkably, the treatment of the culture with BPA caused the accumulation of the reduced form of PDI with concomitant decreases in the levels of the disulfide-linked complexes (Fig. 5*A*). This effect was enhanced as the BPA concentration increased, suggesting that BPA slowed down the reoxidation of PDI in the cells.

Because disulfide bonds that form within a protein or between proteins can alter the mobility of the protein on a gel, we treated the cell lysates with a reducing agent to observe the oxidation status of PDI itself more directly. Indeed, separation of the PEG-maleimide-treated cell lysate on a reducing gel allowed us to identify five distinct bands whose changes in the mobility should reflect changes in the numbers of alkylation on free cysteines in PDI (Fig. 5*B*). As observed clearly in Fig. 5*B*, *lanes 3–6*, BPA treatment increased the level of alkylation on PDI in a dose-dependent manner, indicating again that BPA can slow down the reoxidation of PDI in the cells.

##### BPA Affects the Oxidation Status of PDI in HeLa Cells

We next proceeded to examine whether BPA has an impact on the oxidation status of PDI in the cells. For this purpose, we cultured HeLa cells without DTT treatment in the presence of 400 μm BPA for various times and looked at the oxidation status of PDI in the cells. As observed in Fig. 5, *C* and *D*, BPA caused the reduction of PDI in HeLa cells in a time-dependent manner, indicating that BPA can have a great impact on the redox homeostasis of PDI in the cells. These findings are consistent with the observation that BPA can inhibit the ability of Ero1α to oxidize PDI *in vitro* (see above).

##### BPA Has No Effect on Oxidative Protein Folding Catalyzed by the Prx4-ERp46 Pathway

**FIGURE 6.**
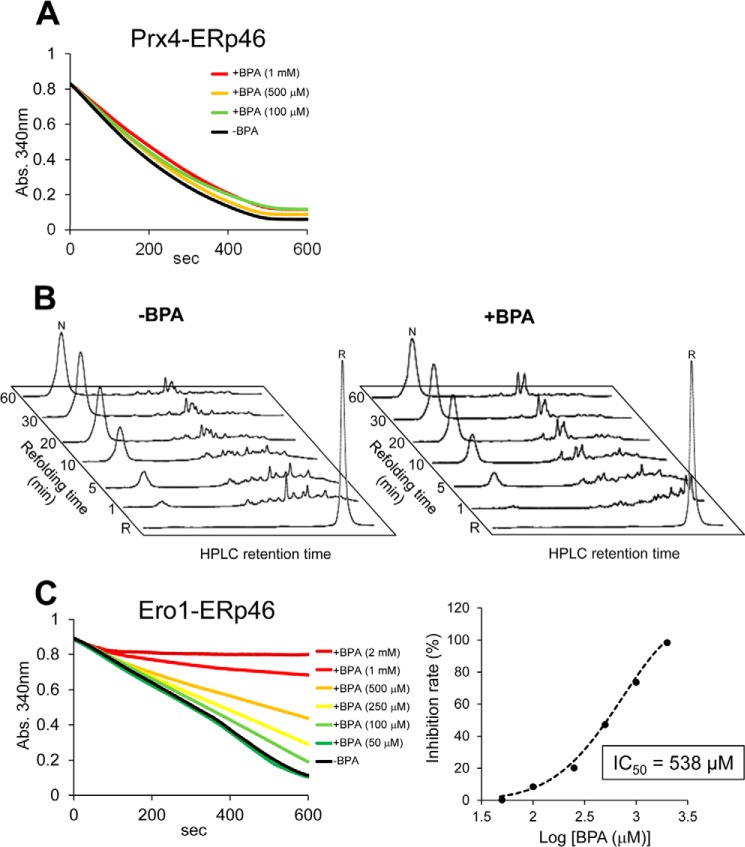
**Oxidative activity of Prx4 and Ero1α toward ERp46 in the presence of BPA.** *A*, oxidative activity of Prx4 (0.4 μm) toward ERp46 in the presence of various concentrations of BPA was assessed by measuring the decrease in *A*_340_, as above. *B*, Prx4-ERp46-driven oxidative folding of BPTI. HPLC profiles indicate the time course of oxidative folding of BPTI catalyzed by Prx4-ERp46 in the absence (*left*) and presence (*right*) of BPA (40 μm). Folding was initiated by the addition of Prx4 (0.1 μm) and ERp46 (1 μm) to reduced BPTI (30 μm) in a buffer containing H_2_O_2_ (200 μm). At the indicated time points, reactions were quenched with HCl and analyzed by HPLC. GSH/GSSG redox reagents were not used in this assay. *C*, *left*, oxidative activity of Ero1α (4 μm) toward ERp46 in the presence of various concentrations of BPA was assessed by measuring the decrease in *A*_340_. *Right*, *plot* of percentage inhibition as a function of BPA concentration determined from the initial velocity of the decrease in *A*_340_.

To further examine the possible role of BPA as an inhibitor of oxidative pathways other than Ero1α-PDI, we analyzed Prx4-catalyzed ERp46 oxidation in the presence of BPA. A BPA titration assay showed little or no effect on Prx4 oxidative activity toward ERp46 (Fig. 6*A*) as well as toward PDI (Fig. 4*A*). In line with this, the addition of BPA had little effect on the HPLC profiles, indicating the time course of BPTI oxidative folding (Fig. 6*B*).

By contrast, BPA had weak but significant inhibitory effect on Ero1α-mediated ERp46 oxidation. The IC_50_ value was 538 μm, more than 10-fold higher than that of Ero1α-mediated PDI oxidation in the presence of BPA (IC_50_ = 41 μm) (Fig. 6*C*). Thus, BPA had the ability to more or less inhibit the oxidative pathways constituted by Ero1α and PDI family members (also see “Discussion”).

##### BPA-induced Conformational Change Revealed by CD

**FIGURE 7.**
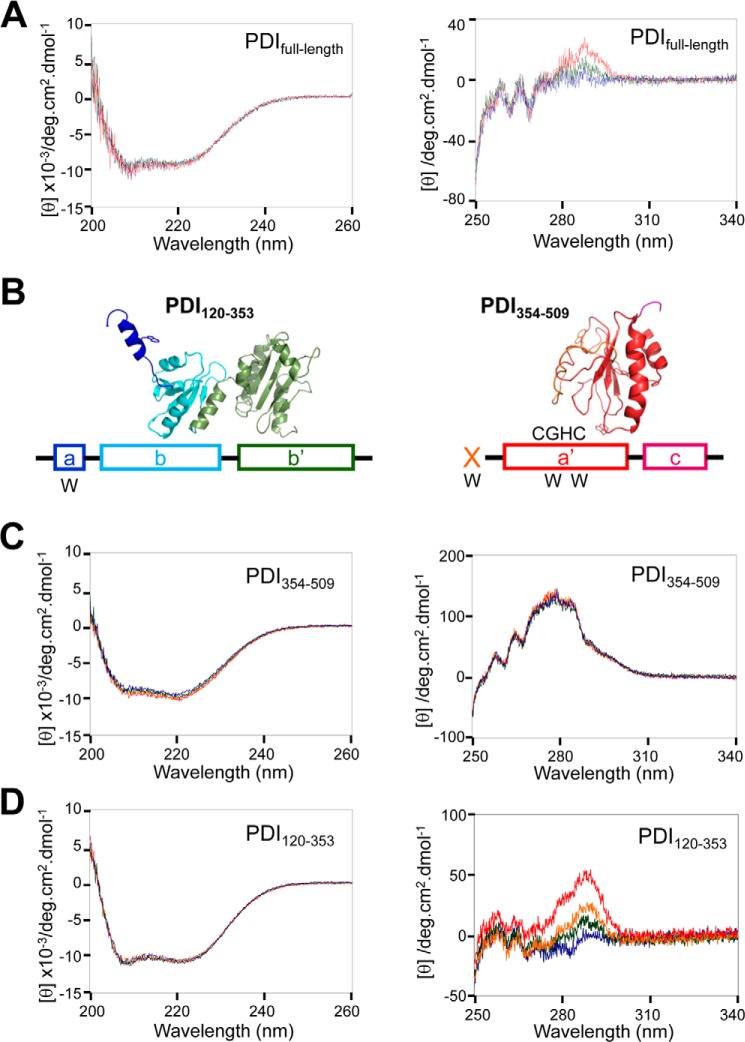
**BPA-induced conformational changes in PDI.** *A*, far-UV (*left*) and near-UV (*right*) CD spectra of PDI_full-length_. The CD curves at BPA/PDI_full-length_ molar ratios of 0 (*blue*), 1 (*green*), and 10 (*red*) are displayed. *B*, structural model of PDI(120–353) and PDI(354–509) predicted using Phyre (31). Tryptophan residues contained in these two fragments are indicated by *W below* the schematic structure. *C*, far-UV (*left*) and near-UV (*right*) CD spectra of PDI(354–509). The CD curves at BPA/PDI(354–509) molar ratios of 0 (*blue*), 0.25 (*green*), 0.5 (*orange*), and 1 (*red*) are displayed. *D*, far-UV (*left*) and near-UV (*right*) CD spectra of PDI(120–353). The CD curves at BPA/PDI(120–353) molar ratios of 0 (*blue*), 0.25 (*green*), 0.5 (*orange*), and 1 (*red*) are displayed.

The functional defects caused by BPA suggest that BPA may induce conformational changes in PDI. To examine this possibility, we measured near-UV and far-UV CD spectra of PDI in the presence or absence of BPA. Although there was no significant change in the far-UV region (Fig. 7*A*, *left*), a slight but significant difference was observed in the near-UV region; a CD signal at ∼290 nm increased upon titration of BPA (Fig. 7*A*, *right*). The results suggest that although BPA had little effect on secondary structure, BPA induced a slight but significant change in the chemical environment around the tryptophan residues of PDI.

To explore which region of PDI undergoes BPA-induced conformational changes, we measured near-UV CD spectra of PDI(120–353), which consists of the C-terminal helix of domain **a** and intact domains **b** and **b′** (Fig. 7*B*, *left*), and that of PDI(354–509), which consists of the **x**-linker and domains **a′** and **c** (Fig. 7*B*, *right*). The former fragment contains only one tryptophan residue (Trp^130^) in the C-terminal region, whereas the latter contains one tryptophan residue in the **x**-linker region and two in domain **a′**.

CD spectra of PDI(354–509) in the near- and far-UV regions were almost identical regardless of the presence or absence of BPA (Fig. 7*C*), indicating that the secondary and tertiary structure of PDI(354–509) were unaltered upon the addition of BPA. By contrast, PDI(120–353) exhibited a marked increase in CD signal at ∼290 nm in proportion to the concentration of BPA added (Fig. 7*D*, *right*), whereas far-UV CD spectra were insensitive to BPA (Fig. 7*D*, *left*). These results strongly suggest that the location and surrounding environment of Trp^130^ contained in domain **a** of PDI(120–353) were greatly affected by BPA despite the lack of large conformational changes in secondary structure.

##### BPA Binds to PDI(120–353) Fragment

**FIGURE 8.**
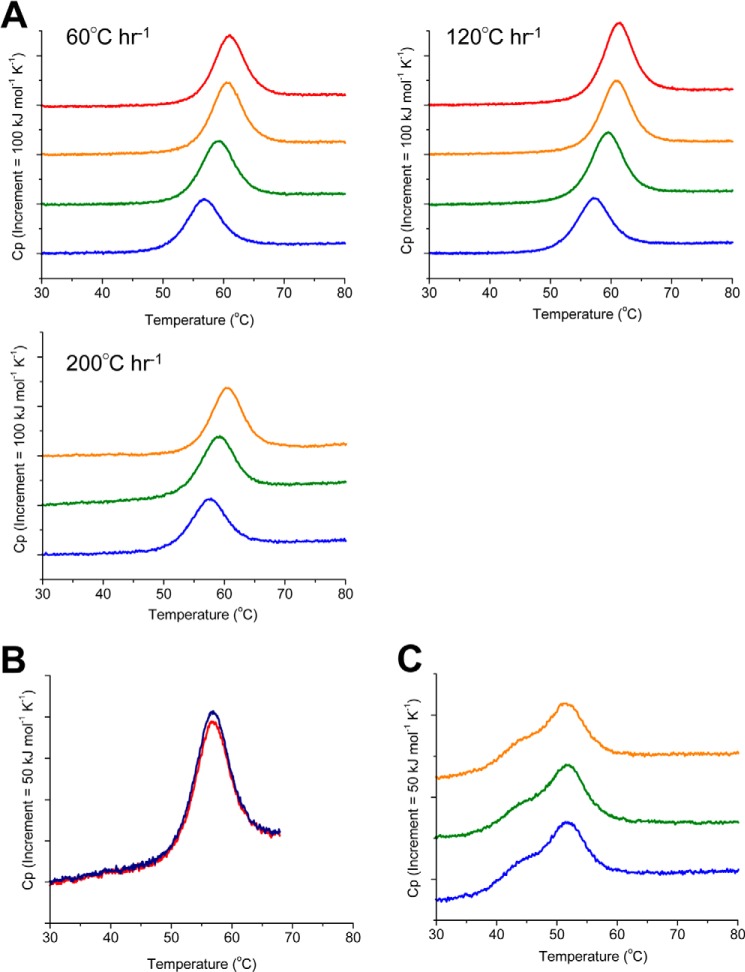
**DSC measurements for PDI(120–353) and PDI(354–509) in the presence of BPA.** *A*, DSC curves of PDI(120–353) at BPA/PDI(120–353) molar ratios of 0 (*blue*), 1 (*green*), 5 (*orange*), and 10 (*red*) are displayed. The DSC curves were measured at a scan rate of 60, 120, and 200 °C h^−1^, respectively. *B*, DSC curve of the second run (*red curve*) was measured for PDI(120–353) at a scan rate of 60 °C h^−1^ immediately after cooling of the first run (*blue curve*). The recovery area of excess heat capacity was 89.3%. *C*, DSC curves of PDI(354–509) at BPA/PDI(354–509) molar ratios of 0 (*blue*), 1 (*green*), and 5 (*orange*) are displayed. The DSC curves were measured at a scan rate of 200 °C h^−1^.

To investigate possible changes in the thermal stability of PDI(120–353) upon the addition of BPA, we performed DSC analysis. Fig. 8*A* shows that the DSC curves for PDI(120–353) were shifted to higher melting temperature (*T_m_*) values in accordance with the BPA/PDI(120–353) molar ratio; the *T_m_* of PDI(120–353) increased by 4.4 °C in the presence of a 10-molar equivalent excess of BPA at a scan rate of 60 °C h^−1^ (Table 1). Similar phenomena were observed at different scan rates of 120 and 200 °C h^−1^ (Table 1 and Fig. 8*A*). Precise analysis of thermal denaturation requires confirmation of its reversibility. To this end, a second run was performed by reducing the temperature after the first run. Successive rescans of PDI(120–353) showed a superimposable curve (89.3%) at a scan rate of 60 °C h^−1^ at pH 7.0 (Fig. 8*B*), indicating that the observed unfolding transition was highly reversible. Changes in unfolding enthalpy by BPA binding are listed in Table 1, suggesting that they are within experimental errors at three different scan rates. Thus, unfolding enthalpy is not drastically affected by BPA binding to PDI.

**TABLE 1:** **Melting temperature (*T_m_*) and the enthalpy of unfolding in accordance with the BPA/PDI(120–353) molar ratio at a scan rate of 60 °C h^−1^ (left), 120 °C h^−1^ (middle), and 200 °C h^−1^ (right)** The Δ*C_p_* value of 1.66 × 10 kJmol^−1^ K^−1^ was used for calculation of Δ*H* at 56.6 °C.

BPA/PDI(120–353)	Scan rate of 60 °C h^−1^			Scan rate of 120 °C h^−1^			Scan rate of 200 °C h^−1^		
	*T_m_*	Δ*H*(*T_m_*)	Δ*H* at 56.6 °C	*T_m_*	Δ*H*(*T_m_*)	Δ*H* at 57.1 °C	*T_m_*	Δ*H*(*T_m_*)	Δ*H* at 57.5 °C
	°*C*	*kJ/mol*	*kJ/mol*	°*C*	*kJ/mol*	*kJ/mol*	°*C*	*kJ/mol*	*kJ/mol*
0	56.6 ± 0.2	318 ± 19	319 ± 20	57.1 ± 0.1	313 ± 4	313 ± 3	57.5 ± 0.1	293 ± 25	294 ± 26
1	59.0 ± 0.5	382 ± 22	342 ± 16	59.4 ± 0.3	375 ± 19	337 ± 14	58.9 ± 0.6	315 ± 27	293 ± 17
5	60.5 ± 0.1	399 ± 21	334 ± 20	60.8 ± 0.2	397 ± 18	336 ± 15	60.5 ± 0.4	354 ± 45	305 ± 52
10	61.0 ± 0.3	376 ± 4	302 ± 2	61.2 ± 0.1	412 ± 10	344 ± 112			

The equation for the relationship between the binding constant of a ligand to a protein and the melting temperature of a protein can be described as follows (45, 46),

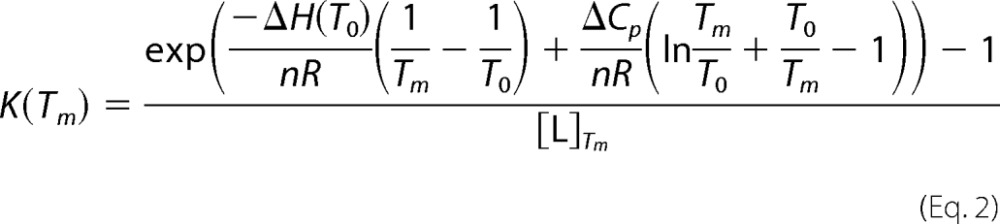
 where *T_m_* and *T*_0_ are melting temperatures in the presence and the absence of BPA, respectively, [L]*_Tm_* is the concentration of unbound BPA at *T_m_*, *n* is the difference in the number of bound molecules of BPA in the unfolded and folded states, *K* is a binding constant, *R* is the gas constant, and Δ*C_p_* is a heat capacity change due to heat denaturation of a protein. The Δ*C_p_* value upon PDI(120–353)-BPA complex formation was estimated to be 1.66 × 10 kJ mol^−1^ K^−1^, using the water-accessible nonpolar surface area and polar surface area of PDI(120–353) calculated from structure prediction with Phyre (45, 46). This equation is applicable only when the enthalpy change of binding is negligible compared with the enthalpy change of protein denaturation (47). In the case of BPA binding, the enthalpy change was negligible within experimental errors as described.

The dissociation constant *K_d_* (1/*K_a_*) between PDI(120–353) and BPA was calculated to be 1.17 × 10^−5^, 1.36 × 10^−5^, and 1.09 × 10^−5^ m^−1^ at a scan rate of 60, 120, and 200 °C h^−1^, respectively (Table 2). The dissociation constants obtained by DSC experiments were similar to that obtained by previous reports (22.6 μm) (6). In sharp contrast, the DSC curves of PDI(354–509) were unchanged even upon the addition of a 5-molar equivalent excess of BPA; DSC curves showed peaks with a *T_m_* value of 51.3 °C, irrespective of the presence or absence of BPA (Fig. 8*C*). These results suggest that only PDI(120–353) enhances the thermal stability due to BPA binding, but BPA does not have any effect on PDI(354–509).

**TABLE 2:** **Determination of the dissociation constant between PDI(120–353) and BPA**

BPA/PDI(120–353)	*K_d_*		
	Scan rate of 60 °C h^−1^	Scan rate of 120 °C h^−1^	Scan rate of 200 °C h^−1^
	*m*^−*1*^		
0	1.17 × 10^−5^	1.36 × 10^−5^	1.09 × 10^−5^
1	1.23 × 10^−5^ ± 0.37 × 10^−5^	1.39 × 10^−5^ ± 0.28 × 10^−5^	1.55 × 10^−5^ ± 0.79 × 10^−5^
5	4.29 × 10^−5^ ± 0.33 × 10^−5^	5.35 × 10^−5^ ± 0.46 × 10^−5^	4.48 × 10^−5^ ± 1.03 × 10^−5^
10	7.02 × 10^−5^ ± 0.84 × 10^−5^	9.14 × 10^−5^ ± 0.41 × 10^−5^	

##### Molecular Diameter and Geometrical Shape of PDI(120–353) in the Presence of BPA

**FIGURE 9.**
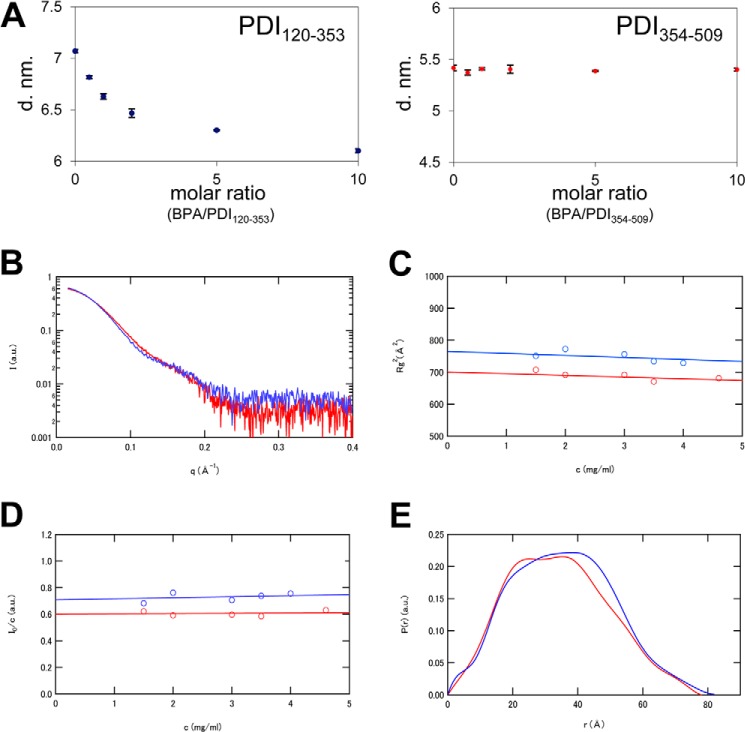
**Size and molecular shape of BPA-bound PDI(120–353) in solution.** *A*, molecular diameters of PDI(120–353) (*left*) and PDI(354–509) (*right*) at various molecular ratios of BPA/PDI. Concentrations of PDI(120–353) and PDI(354–509) were 0.89 and 1.18 mg/ml, respectively. *B*, SAXS curves of PDI(120–353) in the presence (*red*) or absence (*blue*) of BPA. The natural logarithm of zero-extrapolated scattering intensity, ln*I*(*Q*), is shown as a function of scattering angular momentum, *Q. C*, protein concentration dependence of the apparent radius of gyration of PDI(120–353) in the presence (*red*) or absence (*blue*) of BPA. The *continuous lines* denote the least-squares fit of a linear equation to the data. *D*, protein concentration dependence of normalized forward intensity, *I*(0)/*c*, for PDI(120–353) in the presence (*red*) or absence (*blue*) of BPA. The *continuous lines* denote the least squares fit of a linear equation to the data. *E*, pair distribution functions of PDI(120–353) in the presence (*red*) or absence (*blue*) of BPA, extracted from SAXS curves shown in *B*.

To gain further structural insights into the BPA-bound form of PDI(120–353), we performed DLS and SAXS analyses. DLS is often employed to measure the size of target particles (48). Fig. 9*A* shows the molecular diameters of PDI(120–353) and PDI(354–509) measured at different molar ratios of BPA/PDI (ranging from 0 to 10). No significant change was observed in the mean diameter of PDI(354–509) in this range of BPA/PDI molar ratios (Fig. 9*A*, *right*); however, the molecular size of PDI(120–353) markedly decreased upon titration of BPA up to a BPA/PDI molar ratio of ∼5 (Fig. 9*A*, *left*).

To determine the structural parameters and the molecular shape of BPA-bound and -unbound forms of PDI(120–353), SAXS analysis was performed in the presence or absence of BPA. Notably, there was a striking difference between the scattering curves of the PDI(120–353)-BPA complex and PDI(120–353) alone around the *q* value of 0.10 Å^−1^ (Fig. 9*B*), suggesting a significant conformational change upon BPA binding. The apparent radius of gyration, *R_g_*(*c*), and the normalized forward intensity, *I*(0)/*c*, showed only marginal protein concentration dependence in the range of 0–5 mg/ml (Fig. 9, *C* and *D*). The extrapolated *R_g_* value at *c* = 0 was 26.7 Å for the PDI(120–353)-BPA complex and 28.0 Å for PDI(120–353) alone. Thus, PDI(120–353) assumes a more compact structure when it binds BPA. This is consistent with the DLS observations, where the molecular diameter of PDI(120–353) markedly decreased upon the addition of BPA.

To extract information on the geometrical shape of PDI(120–353), we calculated a pair distribution function, *P*(*r*) (Fig. 9*E*). Because *P*(*r*) indicates the distribution of linear distances between every pair of atoms in a particle, the most frequent *r* value and the largest *r* value (*D*_max_) can be determined from this function. The *D*_max_ values were thus estimated to be 82.0 Å for PDI(120–353) alone and 79.5 Å for the PDI(120–353)-BPA complex, corroborating the present finding that BPA binding results in a more compact overall shape for PDI(120–353).

## DISCUSSION

The present study determined that BPA, an endocrine disruptor, markedly inhibited oxidative protein folding catalyzed by the Ero1α-PDI combination *in vitro*. Consistent with the findings, BPA slowed down the reoxidation of PDI in HeLa cells, leading to the accumulation of a large amount of PDI in the reduced form. Thus, BPA can have an enormous influence on the properties of PDI both *in vitro* and in cells.

One of the major effects of BPA on this oxidative system was the rearrangement of the thioredoxin domain of PDI through inward movement of the N-terminal **a** domain, resulting in more compact overall shape. This conformational change probably led to inhibition of the capture of partially folded or misfolded proteins by PDI. Accordingly, the BPTI refolding assay clearly demonstrated that BPA inhibited the late stages of protein folding (and early stages to a lesser extent) from kinetically trapped intermediates to a native state (Figs. 2*B*, 3*A*, and 4*B*). In this context, several lines of evidence suggest the significant plasticity of the **bb′** segment of PDI. Crystal structures of reduced and oxidized forms of human PDI showed a redox-dependent domain arrangement of the **b** domain relative to **b′** (15). In addition, human PDI **bb′** cannot be superimposed onto yeast PDI **bb′** (13, 15). Our study represents the first report that significant rearrangement of the N-terminal **a** domain can also occur when PDI is complexed with BPA. The intrinsically flexible nature of PDI is likely to be advantageous during oxidative folding by enabling PDI to adapt to the size, shape, and folded states of substrate proteins.

Another major effect of BPA on the Ero1α-PDI oxidative cycle could be that BPA prevents Ero1α from binding to the hydrophobic pocket of the PDI **b′** domain and hence inhibits the Ero1α-mediated catalysis of PDI oxidation. We previously revealed that Trp^272^, a highly conserved residue in the protruding β-hairpin of Ero1α, plays a pivotal role during the functional interplay of Ero1α and PDI (22). It is conceivable that phenol groups of BPA compete with this tryptophan residue for binding to the same site in PDI **b′** (25). In support of this, our previous mutational study suggested that His^258^, Gln^245^, and Asn^300^, residues located near the hydrophobic pocket of PDI, were closely involved in BPA binding (25).

Ero1 and PDI have long been known to constitute a major disulfide bond formation pathway, although more recently more than 20 members of the PDI family and more than five enzymes with significant oxidative activity toward PDI family members have been identified in the mammalian ER (18, 19). Thus, the oxidative folding network in mammalian cells is more complicated and diverse than previously thought. Presumably, multiple PDI family members and their oxidation enzymes work in a distinct, cooperative, and sometimes complementary manner to ensure efficient production of multidisulfide-containing proteins (17, 18, 27, 28, 49).

We recently revealed that although Prx4 can oxidize a broad range of PDI family members (27, 50), ERp46 and P5 are the primary partners of Prx4 in cells. The ERp46-Prx4 and P5-Prx4 oxidative pathways are probably dedicated to rapid but promiscuous disulfide bond introduction during the early stages of oxidative protein folding (27, 28). The present study demonstrated that BPA had a marginal effect on Prx4 oxidative activity toward both ERp46 (Fig. 6*A*) and PDI (Fig. 4*A*). Meanwhile, BPA significantly inhibited Ero1α-catalyzed oxidation of ERp46 although with a lower potency (IC_50_ = 538 μm) than that of PDI (IC_50_ = 41 μm) (Figs. 1*C* and 6*C*). The large difference in the effects of BPA on Ero1α- and Prx4-driven oxidative pathways presumably reflects the completely different mode of interaction of these two oxidative enzymes toward PDI family members. Systematic studies indicate that Prx4 oxidizes the thioredoxin domains of PDI family members at almost the same rate regardless of whether they are present on an isolated peptide or as part of an intact protein (27, 28). Ero1α recognizes and oxidizes the tandemly positioned **b′**-**a′** domains of PDI as a minimal element (22, 23). Whereas the BPA binding site of ERp46 remains to be specified, BPA may influence the function of a wide range of PDI family members and hence may alter the redox environment in the ER.

An increasing number of studies have investigated the physiological effects of BPA on mammalian tissues. *In vivo*, BPA induces heat shock protein 90α expression in mouse uterus (51) and induces oxidative stress in rat liver due to decreased antioxidant enzyme activity (52). Also, BPA down-regulates the production and secretion of adiponectin (53), which is widely known as an adipocyte-derived secretory protein that has potent insulin-sensitizing and anti-apoptotic properties (54). Of note, BPA induces ER stress as indicated by the increased expression level of GRP78/Bip, an ER stress marker (55, 56). One possible interpretation is that the inhibitory effect of BPA on PDI activity may cause the accumulation of misfolded proteins in the ER, inducing the unfolded protein response. Thus, BPA may interfere with the synthesis of secretory proteins and their secretion pathways in mammalian cells. In this context, it would be interesting to perform a global analysis of the effects of BPA on secretory protein production and secretion. The present finding of BPA-mediated inhibition of the functional interplay between Ero1α and PDI thus provides feasible insights into the mechanisms by which BPA causes adverse health effects.

## Footnotes

4 The abbreviations used are:

BPA: bisphenol A
DLS: dynamic light scattering
DSC: differential scanning calorimetry
ER: endoplasmic reticulum
Ero1: ER oxidoreduclin 1
PDI: protein-disulfide isomerase
Prx4: peroxiredoxin-4
SAXS: small angle x-ray scattering
BPTI: bovine pancreas trypsin inhibitor
SPR: surface plasmon resonance.
